# Intracellular bioaccumulation of the rare earth element Gadolinium in ciliate cells resulting in biogenic particle formation and excretion

**DOI:** 10.1038/s41598-023-32596-3

**Published:** 2023-04-06

**Authors:** Jana Kohl, Michael Schweikert, Norbert Klaas, Marie-Louise Lemloh

**Affiliations:** 1 Institute of Biomaterials and Biomolecular Systems, University of Stuttgart, 70569 Stuttgart, Germany; 2 SRF AMICA, University of Stuttgart, 70569 Stuttgart, Germany; 3 IWS, Research Facility for Subsurface Remediation (VEGAS), University of Stuttgart, 70569 Stuttgart, Germany; 4 Materials Testing Institute, University of Stuttgart, 70569 Stuttgart, Germany

**Keywords:** Microbiology, Environmental sciences

## Abstract

Ciliates are abundant unicellular organisms capable of resisting high concentrations of metal ions in the environment caused by various anthropogenic activities. Understanding the cellular pathways involved in resistance to and detoxification of elements is required to predict the impact of ciliates on environmental element cycles. Here, we investigated the so far unknown process of tolerance, cellular uptake and bioaccumulation of the emerging rare earth element gadolinium (Gd) in the common ciliate *Tetrahymena pyriformis.* Gd treatment results in the intracellular formation and excretion of biogenic Gd-containing particles. This cellular process effectively removes dissolved Gd from the organic growth medium by 53.37% within 72 h. Based on light and electron microscopic observations, we postulate a detoxification pathway: Cells take up toxic Gd^3+^ ions from the medium by endocytosis, process them into stable Gd-containing particles within food vacuoles, and exocytose them. Stable biogenic particles can be isolated, which are relatively homogeneous and have a diameter of about 3 µm. They consist of the elements Gd, C, O, P, Na, Mg, K, and Ca. These findings broaden the view of metal ion accumulation by protists and are of relevance to understand environmental elemental cycles and may inspire approaches for metal recovery or bioremediation.

## Introduction

Various anthropogenic processes lead to the contamination of our environment with certain metals. Some of these metals are economically important and should be recovered, while others are problematic due to their environmental toxicity or both. The rare earth element gadolinium (Gd) is one of several emerging (rare earth) heavy metals increasingly released into our environment^1–3^. Especially free Gd^3+^ ions are toxic to organisms because they can interfere with or inhibit Ca^2+^ signalling in cells and tissues due to their similar ionic radius to Ca^2+^ ions^3,4^. One application of the complexed form of Gd is as a contrast agent for magnetic resonance imaging (MRI)^3,5,6^. After their application to humans, contrast agents are excreted into the wastewater system and cannot be filtered out by wastewater treatment plants, thus reaching surface water or groundwater^1,3,5^. The stability and fate of various Gd speciation in the environment, in addition to their toxicity effects on aquatic organisms and plants, is the subject of ongoing research^1,7–13^. It would therefore be intriguing to understand better what mechanisms microorganisms have evolved to deal with metal ions such as Gd and whether and how they can remove them from the surrounding environment.

Ciliates (Eukaryota, Alveolata, Ciliophora) are one of the most common protists, and some species are known to tolerate certain concentrations of dissolved (toxic) metal ions in their environment. They can survive even in polluted waters and lakes^14–16^. In addition, ciliates have further characteristics, such as a short generation time, small size and easy handling, which is why they are used as model organisms for (eco-)toxicity studies^17^, as well as a bioindicator for heavy metal pollution^18–20^. The influence of heavy metals on ciliates was mainly investigated in terms of mortality, generation time and cytotoxicity. The main focus of research was on the heavy metals Cd, Cu, Zn and Pb^17^. There is only rare work done on effects of other heavy metals like rare earth elements.

To deal with heavy metal pollution, ciliates possess several metal resistance mechanisms. One detoxification mechanism is the intracellular bioaccumulation of toxic metals, which has been observed in various ciliate species^14,15,21–25^. Bioaccumulation is usually associated with a reduction in metal ion concentrations in the surrounding medium. Under laboratory conditions, for example, ciliates remove about 70% of Pb^2+^ and Cr^6+^ ions from the cell culture medium after 72 h^15^. A direct environmental influence through the formation of intracellular granules in protists has been postulated, e.g. for barium in Lake Geneva, with increased intracellular enrichment correlating with a decrease in the concentration of dissolved elements within the lake^26^. For rare earth elements, it was demonstrated that they have an impact on proliferation rate and cell mobility^27–29^. Metal treatment in *Tetrahymena* also leads to the expression of metallothioneins (MTs)^30–32^. MTs are common proteins with the ability to bind heavy metal ions in the cytosol^20^. The expression of MTs plays a role in bioaccumulation especially for the elements Cd, Cu and Zn^20,30,33^, as well as in tolerance to heavy metals, as shown by experiments with knock-out mutants^31^.

Especially the ciliate species *Tetrahymena pyriformis* (Ciliophora, Oligohymenophorea, Hymenostomatida) is frequently used in (eco-)toxicity studies^25,34,35^. Bioaccumulation is observed in the occurrence of so called small refractive granules inside the cells, which can be detected under unfavourable growth conditions, stress, but also as a detoxification mechanism after metal treatment ^25,36,37^. Furthermore metal-containing material (particles) inside food vacuoles have been observed for example after treatment of *Tetrahymena* with lead, lanthanum, platinum, bismuth, selenium, aluminium and gold^25,28,38–44^. However, little is known about processes of elemental uptake, the pathway of intracellular processing and the properties (consistence and stability) of metal-containing particles.

These earlier discoveries raise the question of how the observation of metal tolerance of the cells, the intracellular metal accumulations and the depletion of metal concentrations in the environment are linked and if this can be transferred to other elements. Concerning intracellular bioaccumulation processes, this work aimed to discover the so far unexplored pathway of Gd bioaccumulation in *Tetrahymena pyriformis.* The question arises if intracellular metal-containing particles are actively formed and controlled by the cells, comparable, e.g. to controlled biomineralization processes, also known for ciliates^45^ or whether this is a secondary effect due to organic molecule interactions with the offered metal ions. This work investigates the influence of the environmentally increasingly relevant metal Gd on *Tetrahymena pyriformis,* aiming to reveal a possible bioaccumulation of Gd as well as a better understanding of underlying intracellular pathways. Here we combine dynamic light microscopic observations with (cryo) electron microscopy and analytical methods to investigate the intracellular pathway of Gd and the biogenic particle formation.

## Results

### Formation of intracellular gadolinium-containing particles in *Tetrahymena pyriformis*

**Figure 1:**
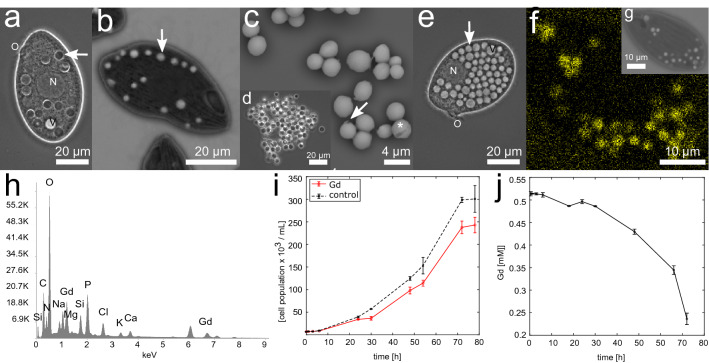
Intracellular Gd-containing particles are formed after Gd treatment. (**a**) Light microscopic phase contrast (LM-Ph) image of *Tetrahymena pyriformis* 4 h after treatment with 0.5 mM GdCl_3_ in 1% PPY medium. Several dark-appearing Gd-containing particles can be observed in the cells (arrow). Characteristics of the cell, like the oral region (O), the nucleus (N), and the contractile vacuole (V), are recognisable. (**b**) BSE-SEM (detection of backscattered electrons in SEM) image of *T. pyriformis* 30 min after treatment with 0.5 mM GdCl_3_ in 1% PPY medium having several Gd-containing particles with bright appearance (arrow). (**c**) BSE-SEM image of excreted Gd-containing particles of *T. pyriformis* 24 h after Gd treatment (0.5 mM in 1% PPY medium). Arrow indicates the small appendix, which is sometimes observed. The asterisk marks the point at which the EDS spectrum was collected (see h). (**d**) LM-Ph image of excreted agglomerates of Gd-containing particles. (**e**) LM-Ph images of a *T. pyriformis* cell in 1% PPY medium (control). Characteristics are the many food vacuoles that appear clear and transparent in LM-Ph filled with dissolved medium components (arrow). (**f**) EDS-mapping of Gd treated cell of *T. pyriformis* in 1% PP medium 24 h after treatment with 0.5 mM GdCl_3_. Shown is the Gd signal (yellow) (see Supplementary Fig. S2). (**g**) BSE-SEM image of the cell shown in f). (**h**) EDS-spectra of excreted Gd-containing particles (asteriskstar in c). (**i**) Comparison of the time [h] dependent growth of population × 10^3^ per mL of *T. pyriformis* in 1% PP medium without (control; black) and with Gd treatment (0.5 mM) (Gd; red). Error bars indicating SEM (standard error of the mean) (N = 3). (**j**) Time [h] dependent depletion of Gd-concentration [mM] in the medium in the presence of *T. pyriformis* cells in 1% PP medium with starting Gd-concentration of 0.5 mM. Error bars indicating SEM (N = 2).

*Tetrahymena pyriformis* grown axenically in an organic medium with dissolved concentrations of 0.05 mM to 3 mM gadolinium-chloride forms numerous Gd-containing particles (Fig. 1, a-d, f + g, Tab. S1). Gd is taken up by the cells, processed intracellularly, and subsequently excreted into the surrounding medium by exocytosis in the form of solid Gd-containing particles. The particles are formed within vacuoles and appear dark in light microscopic phase contrast mode (LM-Ph) (Fig. 1, a, d) and bright when analysing backscattered electrons in scanning electron microscopy (SEM) (indicating elements with higher atomic numbers than surrounding cellular components) (Fig. 1, b + c, g). They are roundish in shape and have an average diameter of about 3.01 µm ± 0.67 µm (standard deviation (SD)), sometimes with a small appendix (Fig. 1, c). A large number of excreted particles can be found in the surrounding medium after adding Gd, typically as sedimented agglomerates (Fig. 1, d). In contrast, cells without Gd treatment display many vacuoles that appear clear and transparent, not containing particles (Fig. 1, e). The particles are formed intracellularly. No extracellular particle formation was evident, as demonstrated by experiments in which the cells are separated through a membrane from cell-free space (see Supplementary Fig. S1). Excreted particles contain Gd (Fig. 1, f+h) as well as C, N, O, Na, Mg, P, Cl, K, and Ca (Si signals from the substrate) (Fig. 1, h), which was detected by energy dispersive X-ray spectroscopy (EDS). Analysing the elemental composition of the particles compared to other cell compartments showed that the particles are rich in Gd, O, Na, Mg, P, K, and Ca (see Supplementary Fig. S2).

Gd-containing particles are formed under a wide range of cell culture conditions. This includes concentrations of organic content in the cell culture medium of 1% PPY (PP: proteose peptone, Y: yeast extract), 1% PP, 0.5% PP and 0.25% PP, pH values between pH 5–10, and it was independent of the addition of GdCl_3_ or Gd(NO_3_)_3_ (for details see Tab. S1). However, particle formation was absent in cells cultured in ultra-pure water (MQ) or if only yeast extract (Y) was used. Gd treatment has a slightly negative effect on cell population growth (Fig. 1, i). Cells treated with GdCl_3_ (0.5 mM) display a similar lag phase as control cells, starting with 6.63 × 10^3^ cells/mL ± 0.17 × 10^3^ cells/mL (standard error of the mean (SEM)) of untreated cells and 6.86 × 10^3^ cells/mL ± 0.18 × 10^3^ cells/mL (SEM) of cells treated with Gd. After 24 h cell numbers of the population increase, while after 72 h, the cell number of Gd treated population is 20% less compared to the population of control cells (Gd treatment: 237.78 × 10^3^ cells/mL ± 13.94 × 10^3^ cells/mL (SEM); control: 298.52 × 10^3^ cells/mL ± 5.93 × 10^3^ cells/mL (SEM)). Growth then stagnates for treated and untreated cell populations. Linked to intracellular particle formation, there is a time-dependent depletion of Gd concentration in the cell culture medium of *T. pyriformis* cells initially treated with 0.5 mM Gd as shown by inductive coupled plasma optical emissions spectroscopy (ICP-OES) (Fig. 1, j). After cultivating the cells with Gd for 72 h, the concentration of Gd in the solution is reduced to 0.24 mM ± 0,01 mM (SEM). Consequently, 53.37% of Gd in the medium is removed compared to the control concentration value (CC) of added Gd (without cells) (CC: 0.51 mM).

### Properties of Gd-containing particles

**Figure 2:**
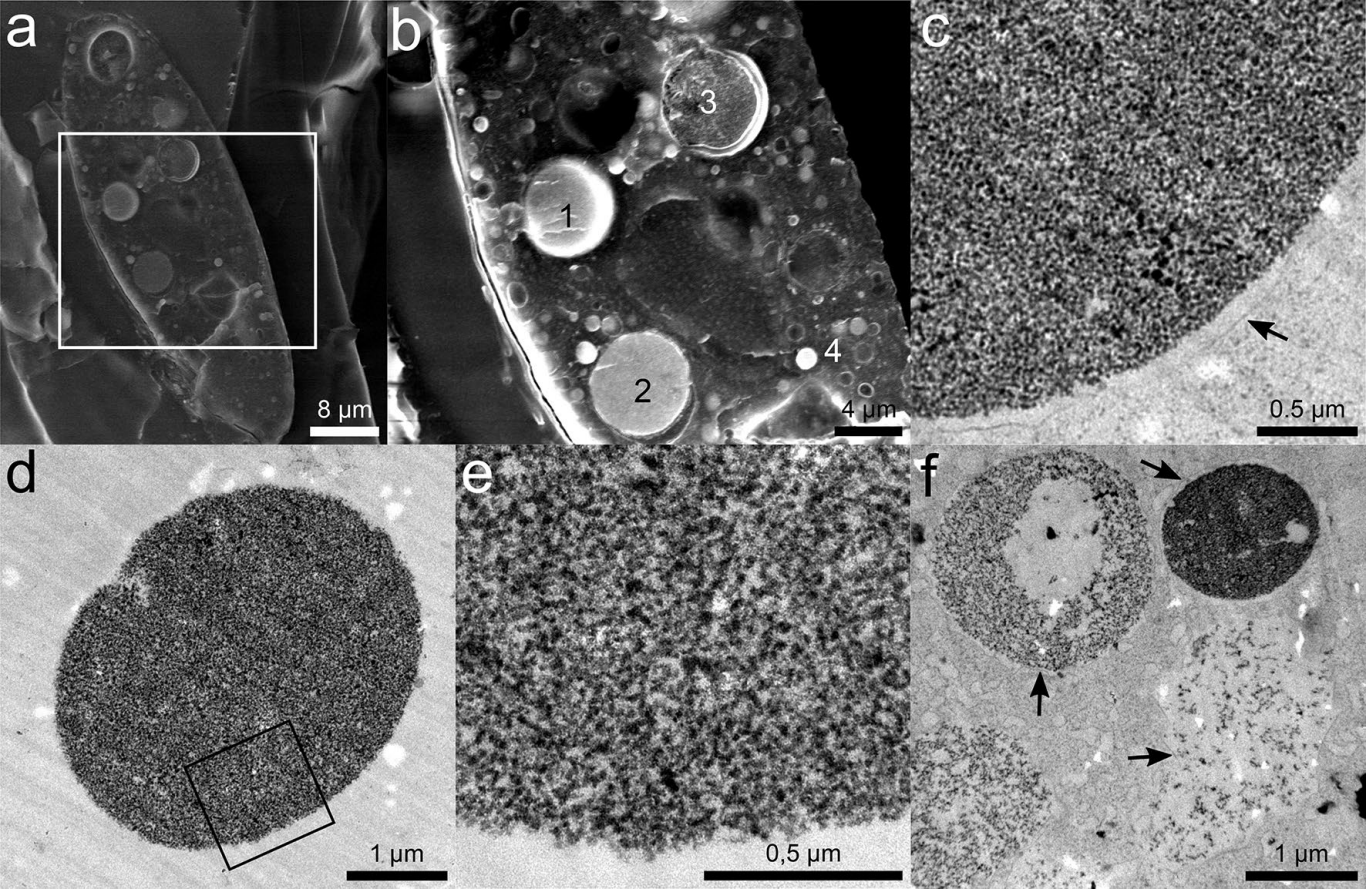
Fine structure and properties of Gd-containing particles. (**a**) SE-cryo-SEM image (detection of secondary electrons in cryo-SEM) image of a freeze-fractured cell of *T. pyriformis* approx. 1.5 h after Gd treatment. (**b**) BSE-cryo-SEM image: Details of indicated area in a. 1: Gd-containing particle; 2: broken Gd-containing particle; 3: broken food vacuole (no Gd); 4: small vesicle (no Gd). (EDS analysis of particle 1 see: S3). (**c**) TEM image of an intracellular electron-dense and fine-structured Gd-containing particle in *T. pyriformis* 2 h after Gd treatment. The particle is surrounded by a membrane (arrow). (**d**) TEM image of an extracellular particle excreted by *T. pyriformis* cell 2 h after starting Gd treatment (membrane absent). (**e**) Details of indicated area in d. Note the fine-structured material network. (**f**) TEM image of membrane-surrounded (arrows) intracellular particles at different stages of denseness in cells of *T. pyriformis* 2 h after Gd treatment. (**a**–**f**) *T. pyriformis* is treated with 0.5 mM GdCl_3_ in 1% PPY medium.

The presence of Gd in the excreted particles was confirmed by EDS (Fig. 1, h). To better understand the inner composition and morphology of the particles, we used SEM analysis of cryo-fixated and freeze-fractured cells as well as transmission electron microscopy (TEM). In freeze-fractured cells treated with Gd, many small vesicles and food vacuoles are visible within the cells (Fig. 2, a + b). Particles containing Gd (Fig. 2, b: 1 and 2) could be distinguished by elemental analysis (EDS) from food vacuoles (Fig. 2, b: 3) and small vesicles (Fig. 2, b: 4) not containing any Gd. It is noticeable that by freeze-fracture, the Gd-containing particles either break out during rupture or are broken apart. Gd, Ca, C, O, and P were detected in the selected area within the particle in the freeze-fractured cells (see Supplementary Fig. S3, a). However, EDS analysis obtained under cryo-conditions revealed less intense signals. The presence of Gd in intracellular particles was additionally confirmed by collecting EDS spectra for ultrathin sections of cells (see Supplementary Fig. S3, b-d) and by analysing the fluorescence signal of Gd superimposed with a light microscopic image (see Supplementary Fig. S4). Intracellular Gd-containing particles are surrounded by a membrane, recognisable in TEM micrographs (arrow in Fig. 2, c, f). In contrast, for extracellular particles, this membrane is absent (Fig. 2, d + e). Particles typically consist of a dense nanostructured material network (Fig. 2, c-f). However, at early time points, e.g. 2 h after Gd treatment, less densely packed material can be observed simultaneously with dense particles (Fig. 2, f).

A procedure to isolate excreted particles from the cell culture mediums was developed by applying centrifugation in the presence of a highly viscous layer of 90% percoll in sucrose. Isolated particles are stable for at least 7 days in cell culture medium (PP), ultrapure water (MQ), tap water, KCl (3 M), ethanol (70%), acetone, percoll (90%), sucrose (2.5 M), NaClO (12%), and they tolerate heat (68 °C) as well as ultrasonic treatment (50/60 Hz, 10 min). However, a significant loss of their roundish morphology indicates that particles are unstable in acids such as HCl (0.5 M) and HNO_3_ (1%), as well as in NaOH (0.5%) and in denaturing SDS (10%).

### Particle formation and excretion

**Figure 3:**
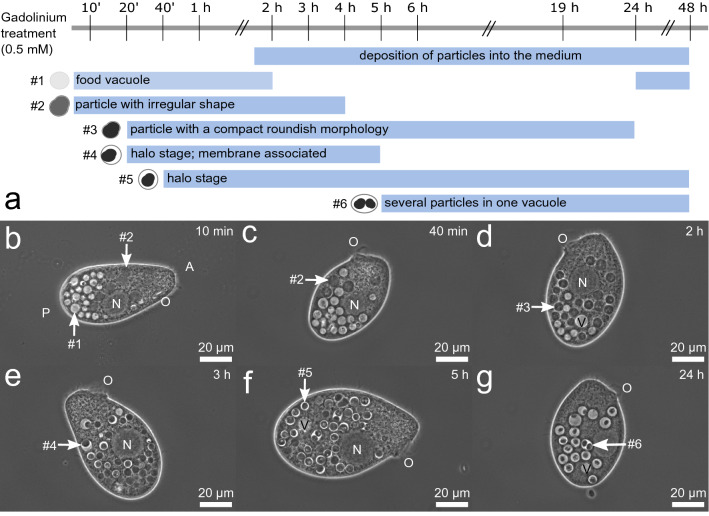
Timeline of the process of intracellular formation of Gd-containing particles in *T. pyriformis*. (**a**) Schematic timeline from 0–48 h after treatment of *T. pyriformis* with 0.5 mM GdCl_3_ in 1% PPY medium. Blue bars mark the time frames in which the respective particle morphology can be observed. (**b**–**g**) LM-Ph images of representative cells of *T. pyriformis* 10 min, 40 min, 2 h, 3 h, 5 h, and 24 h after Gd treatment. Within the cells, many Gd-containing particles, as well as the oral region (O) near the anterior side (A) of the cell, the nucleus (N), and the contractile vacuole (V) near the posterior side of the cell (P) can be observed. Arrows point to particles representative of the described respective particle morphology.

To follow the processes of intracellular particle formation, various cells were examined by light microscopy, revealing different morphological stages of Gd-containing particles occurring as a function of time (Fig. 3). In cells at early time points after Gd treatment (0–2 h), many food vacuoles with transparent appearing contents, as observed in untreated cells, are located near the posterior end of the cell (Fig. 3, b (#1)). Gd treatment results in a reduction of the number of transparent food vacuoles in the cells. While transparent vacuoles are no longer present from about 4 h onwards, they are present again from approx. 24 h after adding Gd. Shortly after Gd addition, vacuoles containing particles with oval irregular shape and, compared to later stages, extended volume are witnessed in the cells (Fig. 3, b-c (#2)). They appear grey in phase contrast, are mainly located near the oral region, and can be identified up to a time of about 4 h. From approx. 20 min after adding Gd, particles with a compact roundish morphology, appearing dark in phase contrast, can be observed over the entire cell body (Fig. 3, d (#3)). Particles with this morphology can be witnessed almost over the whole timeline, although their number dominates at time points from 2 to 4 h after Gd addition. This compact particle morphology can be distinguished from particles with visible vacuolar lumen around them, called the halo stage. The vacuolar lumen surrounding these halo stage particles can be more or less pronounced. Between 40 min and 4 h after Gd addition, compact particles are found mainly in close or partial contact with the vacuole membrane (Fig. 3, e (#4)). Whereas at later time points (5 h- 24 h), particles entirely surrounded by a visible vacuolar lumen are more abundant (Fig. 3, f-g (#5)). Vacuoles containing more than one particle can be observed 5 h after starting Gd treatment (Fig. 3, g (#6)). The size of the particles decreases from particles with irregular shape and extended volume (#2) to compact particles (#3), with a further reduction in size from compact particles (#3) to particles in the halo stage (#4 and #5) (see Fig. 3, b-g). Excretion of particles, except for early particles with an oval irregular shape (#2), at the cytopyge region (at the posterior side of the cells) starts approx. 2 h after the addition of Gd. It takes a few seconds (video S5) and occurs at intervals of a few minutes (Fig. 3, a). To investigate whether excreted particles could be ingested by the cells, excreted particles were separated and offered to untreated cells, resulting in a small number of Gd-containing particles inside these cells after 24 h (see Supplementary Fig. S6).

It is important to mention that the Gd-containing particles clearly differ from granules regularly found in ciliates. Small granules with a diameter of about 0.6 µm (see Supplementary Fig. S7) appear refractive in LM and bright in BSE-SEM (Fig. S7, a and b + c, respectively). They are detected only under conditions where the cells are further stressed (e.g. pH above 9) or starved (fewer organic media content) as well as when treated with 35 mM CaCl_2_ in the medium, with and without simultaneous Gd treatment (see Supplementary Fig. S7, b + c and f, respectively). An overview of the conditions under which the granules are found is provided in Tab S1. Small granules consist mainly of the elements C, O, P, Na, Mg, and K, sometimes with S and Cl (see Supplementary Fig. S7, d), and also Ca and Ga can be deposited (see Table S1). It is also worth mentioning that treatment of *T. pyriformis* cells with additional calcium (35 mM CaCl_2_) did not lead to a particle formation comparable to Gd treatment (see Supplementary Fig. S7, c). However, simultaneous treatment with Ca and Gd results in an increased Ca content inside formed Gd-containing particles (see Supplementary Fig. S7, e–f). Note, that the 1% PP medium itself has a baseline calcium concentration of about 0.16 mM.

To investigate if a Gd-containing precipitate is formed within the organic cell culture medium independent of cellular activity and prior to cellular uptake of Gd, different conditions were examined with regard to the formation of a precipitate (Tab. S1). A fluffy precipitate in the medium appears at pH 9 and 10 (both 0.5 mM Gd) and at pH 7 if Gd concentration is increased to above 1 mM simultaneously. Precipitate formation increases with increasing Gd concentration as well as decreasing concentration of organic compounds in the medium (Tab. S1). The formation of a precipitate is independent of the presence of the cells. Precipitate consists of the elements C, O, Na, P, Cl, K, Ca, and Gd, as shown by EDS (see Supplementary Figure S8). The formation of precipitate leads to a decrease in the dissolved content of Gd in the medium. This was confirmed by analysing the cell-free solution in ICP-OES after the precipitate was removed by filtration. The remaining soluble Gd concentration was decreased to 0.9 mM at conditions with pH 7 and an initial addition of 4 mM GdCl_3_, while no remaining soluble Gd was detectable at pH 10 with 0.5 mM GdCl_3_. Except for the conditions mentioned above, no precipitate is formed. This applies in particular to the conditions for which particle formation is described (0.5 mM Gd, 1% PP, pH 7).

## Discussion

The findings of this research prove that ciliate cells are capable of removing soluble Gd from their surrounding environment by an intracellular bioaccumulation process that results in the excretion of stable biogenic Gd-containing particles. It was proven that this is a biological process and not a chemical precipitation and a cellular pathway of particle formation is postulated. This bioaccumulation process is most likely beneficial for the cells concerning detoxification and most likely not specific to Gd in particular.

Although Gd is used in various fields of human activity, e.g., materials and electronics, alloys, or optical components, research data on the occurrence of Gd in different ecosystems are still limited^3^. An annual emission of 4 tons of Gd has been estimated for Germany^46^. At the same time, the stability and fate of various Gd speciations in the environment and ecotoxicological effects are the subjects of ongoing research^1,7–13^. Intracellular bioaccumulation of Gd in *Tetrahymena pyriformis* was observed in our experiments in organic media after Gd salt treatment at concentrations of 0.05 mM to 3 mM, at a pH range from 5 to 10, independent of the Gd compound (chloride or nitrate) offered. In the case of the use of Gd in contrast agents, Gd concentrations in a range about 5 to 6000 mg/L [0.03 mM to 38 mM] are found close to the medical application to the patient, e.g. in the urine of patients^47,48^, which is in the range of the concentrations that results in Gd-containing particle formation. After processing and dilution, reported values for Gd in our environment are reduced to a range between 0.35 to 80 µg/L^13^ [2.2 nM to 0.5 µM], thus the formation of Gd-containing particles probably plays a subordinate role in freshwaters but is of interest for treatment of wastewater or for element recovery. We expect that the mechanism described here resulting in intracellular particle formation and excretion is not limited to Gd. For other heavy metal contaminants, like the rare earth element lanthanum high concentrations about 52 mg/L [0.37 mM] were measured in the River Rhine^49^. Further for the elements As and Cr, both, the tolerance and bioaccumulation in Ciliates as well as environmental concentrations are in a µM range^24,31,50–53^.

Although adding 0.5 mM GdCl_3_ to the cell culture medium has a slightly inhibitory effect on the growth of *T. pyriformis*, the cells removed 53.37% of the dissolved Gd within 72 h of exposure. An impact of REE on cell growth was also reported for *T. shanghaiensis*. Low concentrations (0.125–0.25 mM) of the rare earth metals lanthanum, samarium and yttrium stimulated cell population growth. In comparison, higher concentrations (0.34 mM in Gd case) inhibit cell population growth^29^. Reduced cell growth could be explained by the energy the cells had to expend for detoxification mechanisms^25^. After 72 h, it is very likely, that depletion of essential nutrients is the reason for the observed stationary growth phase. For comparison, in cell growth experiments with *T.* *thermophila* treated with 0.3 mM GdCl_3_ within a cell culture medium with significantly lower organic content (0.05% vs 1% in our experiments), cells died after 72 h^27^. This is in accordance with prior studies that reported that the organic medium content was essential for detoxification processes and correlates with toxicity effects^28^.

Gd-containing particles formed by the cells mainly consisted of the elements Gd, C, O, P, Na, Mg, K, and Ca. It is likely that the material of the particles was phosphate compounds combined with calcium and trace elements in an organic matrix because of intracellular Gd^3+^ precipitation together with biomolecules. To characterize the chemistry and properties of the particles in detail, further studies are required. Previous studies have investigated the influence of heavy metals on cell morphology and viability of *Tetrahymena*^25,54,55^. Interestingly, for the treatment with the elements lead, lanthanum, platinum, bismuth, selenium, aluminium and gold, the appearance of food vacuoles filled with electron-dense material, comparable to the intracellular particles found in this study, was mentioned^28,38–44,56–58^. However, as in the mentioned studies, no detailed investigations on the bioaccumulation pathway of these metals were made. It remains to be investigated whether these elements follow the same pathway reported here for Gd.

On the other hand, the occurrence of smaller, electron-dense, and in LM refractive granules was described in cells during the stationary growth phase and unfavourable growth conditions^36,37,42,59,60^. We want to emphasize that our results clearly show that a distinction must be made between particles containing metals (as in our case Gd) and having a size of about 3 µm on the one hand and smaller granules with a size of about 0.6 µm on the other hand. The small granules reported in this work occurring during the stationary growth phase, under unfavourable growth conditions or after further CaCl_2_ treatment are consistent with prior literature data on granules that were assumed to play a role in ion regulation and storage, especially that of Ca^2+^ ions^37,61^. During metal treatment of the cells, heavy metals could be deposited in the granules along with, indicating a role in detoxification as well^22,35–37,39,60^.

**Figure 4:**
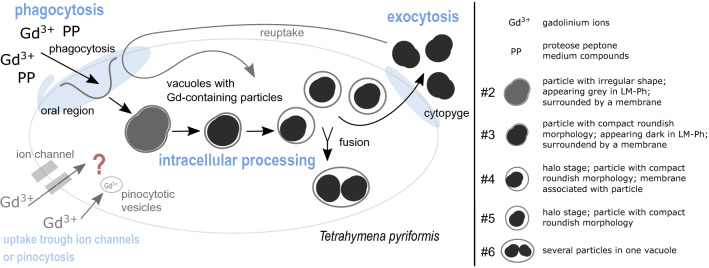
Postulated pathway of the formation of Gd-containing particles in *T. pyriformis*. We postulate the main uptake route of gadolinium (Gd) takes place at the oral region via phagocytosis into food vacuoles. The gadolinium ions (Gd^3+^) are either present as free ions or possibly associated with organic medium compounds of proteose peptone (PP). The Gd-containing content inside the food vacuoles is intracellularly further processed and condensed, which explains the different appearing particle morphologies (#2-#6). We postulate that particles with irregular shape and extended volume (#2) are processed into particles with compact roundish morphology (#3), for which, in turn, the vacuolar lumen becomes visible due to further condensation (#4/#5). Some food vacuoles fuse, creating food vacuoles with more than one particle (#6). An excretion of particles (egestion) occurs through exocytosis at the cytopyge. An ingestion of excreted particles through phagocytosis into food vacuoles is possible at the oral region. An alternative uptake of gadolinium ions through ion channels in the cell membrane or pinocytosis into small pinocytotic vesicles is conceivable and cannot be excluded based on our recent research.

The combination of live cell imaging with electron microscopy allowed us to follow the bioaccumulation and a pathway of intracellular formation of Gd-containing particles in *T. pyriformis* is postulated (Fig. 4). The formation and further processing of the Gd-containing particles take place intracellularly in vacuoles, likely food vacuoles. The particles move through the cells along the typical pathway for food vacuoles during cyclosis, from the anterior end of the cell near the oral region to the posterior end of the cell, to be excreted as stable particles by exocytosis at the cytopyge region. This is most likely a detoxification mechanism by which dissolved Gd^3+^ ions were ingested and bound in stable, after excretion, extracellular particles, resulting in a reduction of toxic Gd in the cellular environment and thus resulting in Gd resistance of the cells at least up to concentrations of 3 mM.

In ciliates, particulate substances are usually taken up via phagocytosis, while dissolved substances and ions are transported via channels through the plasma membranes into the cytoplasm^62,63^. Our results suggest an uptake of Gd via phagocytosis either associated with organic medium compounds of proteose peptone or in the form of free ions. There are several suggested roles for the organic compound proteose peptone in this process: Besides providing essential nutrients, organic media compounds can stimulate food vacuole formation^63,64^, and for REE, it is known that they can speciate with organic medium compounds^11,65^. In general, the toxicity of heavy metals decreases with increasing organic content in the medium^25,28^.

The ICP-OES results of the precipitate control experiments show that Gd is present as soluble ions in 1% PP media at pH 7 with adding 0.5 mM GdCl_3_. However, a Gd-containing precipitate within the cell culture medium, independent of the cell presence, can be induced, e.g., by high pH values. This was expected since, based on the solubility product of Gd(OH)_3_ (K_L_ = 1.8 × 10^–23^ [mol^4^/L^4^]), the added concentration of 0.5 mM (78.6 mg/L) is above the maximum solubility of Gd^3+^ of about 2.8 ng/L at pH 10. However, organic medium components might further affect the solubility of Gd. Nevertheless, the formation of Gd-containing particles was independent of such a precipitate. This finding contrasts prior studies for other elements, where uptake of precipitate was connected with the formation of particle-filled food vacuoles^28,39,42,58^. It illustrates the importance of careful selection and monitoring of cell culture conditions that can alter the speciation of heavy metals and bioavailability^11,65–68^.

In principle, also an uptake of Gd via pinocytosis or other transport mechanisms across the membrane, e.g. through ion channels, is possible and cannot be excluded based on our research (Fig. 4). An uptake of Gd^3+^ ions via calcium transporters would be conceivable, due to similar ionic radius. However, it must be mentioned that treatment of the cells with increased calcium concentrations leads to a different reaction of the cells than Gd treatment. Gd treatment results in particle formation, whereas after Ca treatment, no such particles were observed but granules. Studies describing pinocytosis in *Tetrahymena* are scarce and not sufficiently up-to-date to be able to make precise statements about the possible uptake of Gd^63,69–71^. There is evidence that substances taken up via pinocytosis (coated pits) are later found in lysosomes and old food vacuoles^71^. However, the uptake pathway of Gd via pinocytosis can only be a secondary pathway, as the volume or formation rate of the pinocytosis vesicles would not be sufficient to form the volume of food vacuoles filled with Gd-containing particles within a short time^62,63,71^.

The different morphological stages of the intracellularly formed Gd-containing particles and their respective clustered occurrence at certain time points after starting Gd treatment suggest that the Gd-containing content of the food vacuoles is processed before excretion at the cytopyge. With time the size of particles decreases and a darker appearance with the occurrence of a visible vacuole lumen, in phase contrast, as well as a denser nanostructured material, witnessed in TEM, indicate a condensation of the metal–organic phase within the food vacuoles. Comparable morphological stages with and without visible vacuole lumen (halo stage) were described for food vacuoles containing a mix of bacteria and India-Ink particles^72^ as well as in cells cultivated in the presence of lead^42^. However, the mechanisms inside food vacuoles resulting in precipitation of Gd and solid particle formation still have to be identified, while pH alterations, the expulsion of water from the vesicles, enzymes or proteins with the ability to bind heavy metals such as metallothioneins (MTs) (known to interact, e.g. with La^3+^ ions^32^ and other heavy metals^31^) might be involved in the process. Thus, similar questions will have to be addressed as for vesicular concentration of ions in processes resulting in the formation of biominerals in biomineralization^45^.

Vacuoles in Gd treated *T. pyriformis,* having two Gd-containing particles, were most likely formed by vesicle fusion as known, e.g. for food vacuoles^72^. The Gd-containing particles formed are excreted by exocytosis at the cytopyge, comparable to the egestion of non-digestible pellets^73^. As shown for separated particles, they can be reingested by the cells. Due to Gd depletion (e.g. after 24 h), particle formation is reduced, and newly formed food vacuoles are filled only with medium components again.

Since Gd-containing particles are excreted and formed in vacuoles (primary function in digestion), these metal-containing particles do not appear to have further biological function than detoxification. This is in contrast to the small granules mentioned above, which are involved in the regulation and storage of ions^37^, and in contrast to barium- or strontium-containing particles in Müller vesicles, which act as gravity sensors in some ciliates^74,75^.

We assume that in *Tetrahymena,* the described pathway of uptake and processing of toxic ions into the form of stable and less toxic particles is a general detoxification mechanism for certain heavy metals. This mechanism benefits the cells themselves as they actively change the chemical composition of their surrounding medium. At a larger scale, such processes may change the concentration of dissolved elements in the local environment as suggested, e.g. for barium in Lake Geneva by the formation of intracellular mineral inclusions, denoted as ‘micropearls’, in other protists^26^, or they may influence mineralogical diversity in lakes as described for microbial biomineralization processes as known for the connection of water chemistry and mineralogical diversity with microbial biomineralization activity^76^. Interestingly, ciliates are frequently found also in polluted waters and lakes^14–16^. Although the significance of such processes for elemental cycling has to be determined, the impact of ciliates may have been underestimated to date, especially considering their abundance.

Moreover, this unique bioaccumulation and particle formation process is of importance for research on bio-inspired detoxification or recycling of heavy metals. The here-reported excreted particles are sufficiently stable and can be isolated from the medium, thus allowing for removing Gd from the solution. We assume that the process of particle formation and excretion could also take place with other environmentally relevant elements, which presumably makes it possible to apply the process on a wide scale. In contrast to most known bioremediation processes, there would be no need for chemical desorption steps, as it is required, e.g. for microalgae where metal-ions are adsorbed to their extracellular polymers^77^ and ciliates can be reused in contrast to living microalgae exhibit intracellular bioaccumulation, as they have to be lysed and fractionated to separate metal-containing compartments^78,79^. Finally, this work will contribute to the field of materials science, particularly regarding the fast and low-energy biogenic production of uniformly sized particles.

## Materials and methods

### Cell culture and Gd treatment

Strains of *Tetrahymena pyriformis* (Strain 1630/1W) (Culture Collection of Algae and Protozoa (CCAP)) were maintained axenically in a culture medium of 1% Proteose-Peptone-Yeast-Extract (PPY) (10 g/L Proteose Peptone (PP), 1.25 g/L Yeast-Extract (Y)) or 1% PP, in batch cultures under indirect daylight with a 12:12 h light–dark cycle at 20 °C. Always cells approx. 4-5 days after being transferred into fresh medium (once a week) were used for comparable experiments.

Experiments were performed using different test mediums: 1% PPY medium, 1% PP medium, Yeast-Extract medium (1.25 g/L Y), and MQ medium (ultrapure water). 1% PPY and 1% PP had an unadjusted pH of about 7. 1% PP medium at pH 4–6, and 8–10 was adjusted by adding HCl, respectively, NaOH.

For experiments with a test medium other than culture medium, the cells were washed twice according to the following protocol: 3 mL from a *T. pyriformis* culture were taken and suspended in 9 mL of test medium and then centrifuged at 250 × g for 1 min. The supernatant was removed, and the pellet was resuspended in 3 mL of test medium. The cells were washed under sterile conditions.

Gd treatment experiments were carried out in a total test volume of 3 mL using solutions of gadolinium-chloride (GdCl_3_ * 6 H_2_O (Alfa Aesar)) or gadolinium-nitrate (Gd(NO_3_)_3_ * 6 H_2_O (Alfa Aesar)). To prevent interactions of the cells with precipitate flocs occurring at concentrations above 1 mM GdCl_3_, experiments were, if not otherwise described, carried out with Gd concentrations of 0.5 mM, at which such precipitate flocs were not observed. The impact of calcium was tested in 1% PP Medium with 35 mM CaCl_2_ with and without simultaneous Gd treatment (0.5 mM GdCl_3_). In experiments without additional adding of calcium, calcium concentrations were about 0.16 mM (ICP-OES measured).

Cell growth experiments of Gd treated (0.5 mM GdCl_3_), and control cells were performed in 1% PP medium. Cells 5 days after the last transfer into fresh medium were diluted tenfold in fresh medium. Cell numbers were determined using a Sedgewick-Rafter chamber. Ten time points within 0–78 h after Gd-treatment, respectively after transfer into fresh medium for control experiments, were observed by counting 27–36 squares, by which the arithmetic mean was calculated. The experiment was performed in triplicate. The arithmetic mean, standard deviation (SD) and standard error of the mean (SEM) were calculated from triple experiment data. For cell counting, cells were immobilized by adding 3 M KCl to a final concentration of 0.6 M.

### Light microscopy

For light microscopical investigations, a Zeiss Axiovert 200 M microscope equipped with a plan-Neofluar 1.3 Oil 40 × objective was operated in phase contrast and a plan-Aprochromat 1.4 Oil 63 × objective in differential interference contrast (DIC) mode. A blue filter was used in the beam path. Images were recorded with a Zeiss AxioCam MRm Camera and processed using the AxioVision (Rel. 4.8) software.

A small volume was taken from the experimental set-up and transferred to an object slide. It should be noted that the cells are shown squashed in the images due to the pressure of the cover glass onto the cells. As a result, the cells are immobilised, and the cell organelles, such as food vacuoles, can be clearly imaged. To create a time series, several cells (in 1% PPY medium) were examined at different time points (e.g., 1 h, 2 h, etc.) after adding the Gd solution. At least two replicates were performed for each time point of the experiment, and about 10 cells were examined per experiment.

Contrast and brightness adjustments were performed using ImageJ software (ImageJ 1.53f.). Drawings were created using Inkscape (1.0.1), as well as the insertion of scale bars and the creation of figures.

### Electron microscopy

For SEM analysis, cells were washed with MQ water in a cell culture insert with 3 µm pore size and transferred and air-dried onto a silicon wafer.

For cryo-SEM analysis, 18 mL of a cell suspension from each experimental set-up was harvested by centrifugation in an oil test centrifuge at 200 × g for 2 min. Concentrated cells were mixed with dextran (final concentration of 5%) and concentrated again by centrifugation (at 114 × g for 2 min). The cells were placed between two metal disks (0.05/0.25 mm) HPF carriers and were high-pressure frozen (HPF) using the cryo-immobilisation device Leica EM ICE. Samples were stored in liquid nitrogen and transferred to a Leica ACE 600 cryo sputter coater under cryo conditions (Leica VCT 500). The specimen was freeze-fractured with the built-in cryo knife at -150 °C. Freeze-etching was performed at the maximal rate of up to -100 °C for 10 min with a vacuum of 1*10^–6^ mbar. After freeze-etching, the sample was sputter-coated with an approximately 8 nm thick carbon layer.

A Zeiss EVO 15 scanning electron microscope equipped with the Smart SEM software and, optionally, a Leica cryo-stage was used to examine the cells. Images were obtained at 15 kV and 300 pA. For cryo-SEM, the cryo-stage temperature was kept at -150 °C. Images were obtained at 12 kV, and 50 pA. Either secondary electrons (SE) or backscattered electrons (BSE) were detected.

EDS signals were detected at the respective parameters described above at point or area loci for 200 s using EDAX EDS detector and APEX software. EDS analysis of ultra-thin sections prepared for TEM analysis was performed at 20 kV and about 400 pA.

For TEM analysis, cells were harvested and high-pressure frozen as described for cryo-fixation for SEM. The only difference was applying a thin layer of soy lecithin to the metal disks before loading the sample. After HPF, freeze-substitution (FS) (Leica AFS) was performed. The freeze substitution solution was 0.1% uranyl acetate with 1% OsO_4_ in 100% anhydrous acetone. FS programme: 12 min at -90 °C followed by rewarming at 4 °C per h to 0 °C. Samples were washed 2 × for 15 min each with 100% anhydrous acetone, embedded in Spurr’s resin in stages overnight, and placed in the heating oven to polymerize at 60 °C overnight. Serial sections were made with an ultramicrotome (Ultracut UCT, Leica) with a diamond knife. Sections obtained were approximately 90–100 µm thick and were transferred to previously prepared grids coated with Pioloform (0.8% in chloroform). Cells were monitored with the Zeiss EM10A transmission electron microscope, while images were recorded with a 1K CCD Slowscan camera (TRS).

### Separation and isolation of particles and examination of their properties

A procedure for cell-free isolation of the particles was established for a better yield besides separating particles (see supplementary). Particles were isolated by centrifugation through a viscous layer of percoll. Particle agglomerates at the bottom of the culture dish from experimental set-ups with *T. pyriformis* in 1% PP medium containing 0.5 mM GdCl_3_ after 24 h cultivation were separated loose by pipetting. The retrieved volume was gently added on top of a 3 cm layer (15 mL tube) of a percoll-solution (90% percoll in 2,5 M sucrose) and centrifuged at 100 g for 30 s – 1 min. The pellet was resuspended in MQ water. 2–3 pellets were merged and gently added on top of a second percoll-layer (3 cm in 15 mL tube) and centrifuged at 100 g for 3 min. The pellet was resuspended and washed in 3–6 mL of MQ water and centrifuged at 100 g for 2 min. The received pellet was resuspended and washed in acetone. The obtained fractions of isolated particles were free of cells and percoll residues.

For stability tests, the isolated particles were resuspended back in MQ-water as blank. Isolated particles were incubated in different solutions for several days and examined for morphological changes in LM and in SEM with EDS (preparation: briefly washed in MQ and air-dried). Used solutions were: 1% PP medium, MQ-water, tap water, KCl (3 M), ethanol (70%), acetone, nitric acid (HNO_3_) (1%), HCl (0.5 M), NaOH (0.5 M), sodium hypochlorite (NaClO) (12%), percoll (90%), sucrose (2.5 M) and SDS (10%). In addition, separated particles were treated for 10 min in an ultrasonic bath (50/60 Hz) and stored at 68 °C in MQ for several days.

### Inductively coupled plasma optical emission spectrometry (ICP-OES)

Before measurement, all samples (dual determination) for ICP-OES analysis were filtered through a 0.2 µm membrane. Samples containing cells were additionally filtered by centrifugation in a 1 µm cell-culture insert (Sarstedt) at 3150 g for 2 min beforehand.

The Gd-determination was analogous to DIN EN ISO 11885^80^, using the Gd-lines at 335.047 nm and 342.247 nm. The samples were diluted with 1% HNO_3_ by a factor of 10 to minimize the influence of matrix effects caused by the medium used.

Quantitative measurements of Gd-concentrations were carried out in duplicates on approaches with cells of *T. pyriformis* of 5-day-old cell culture (in 1% PP) (total volume 7 mL)) and treated with GdCl_3_ (0.5 mM). Several controls were also measured: control medium (CM): 1% PP medium; Control Concentration (CC) 0.5 mM GdCl_3_ in 1% PP medium (0 h, 24 h); negative control (NC): *T. pyriformis* in 1% PP; positive control (PC): Gd-solution (5 mM tenfold diluted). To better understand the conditions precipitate is formed within the organic medium, concentrations of 1% PP at pH 10 with GdCl_3_ (0,5 mM) and 1% PP at pH 10 and 1% PP with GdCl_3_ (4 mM) were measured. Removal efficiency in % was calculated with the following equation: ((CC (0 h))—value at 72 h) / CC (0 h). ICP-OES data were obtained in mg/L and converted to mM (M(Gd): [157.25 g/mol]).

## Supplementary Information

Supplementary Video 1.

Supplementary Information 1.

## Data Availability

The datasets generated during and/or analyzed during the current study are available from the corresponding author on reasonable request.
