# Supplementary material for: Intracellular bioaccumulation of the rare earth element Gadolinium in ciliate cells resulting in biogenic particle formation and excretion
Source: Sci Rep. 2023 Apr 6;13:5650. doi: 10.1038/s41598-023-32596-3 (PMC10079679; doi:10.1038/s41598-023-32596-3)
Supplement: Supplementary file 2 — Supplementary Information 1. [file 41598_2023_32596_MOESM2_ESM.pdf]

Supplementary Information:
Intracellular bioaccumulation of the rare earth element Gadolinium in ciliate cells resulting in biogenic particle formation and excretion.

Jana Kohl[a], Michael Schweikert[ab], Norbert Klaas[c], Marie-Louise Lemloh[bd*]

[a]University of Stuttgart, Institute of Biomaterials and Biomolecular Systems, 70569 Stuttgart, Germany
[b]University of Stuttgart, SRF AMICA, 70569 Stuttgart, Germany
[c]University of Stuttgart, IWS, Research Facility for Subsurface Remediation (VEGAS), 70569 Stuttgart, Germany
[d]University of Stuttgart, Materials Testing Institute, 70569 Stuttgart, Germany
*Corresponding author: Marie-Louise Lemloh; marie-louise.lemloh@amica.uni-stuttgart.de

Scientific Reports 2023

Supplemenary Methods

Fluorescence microscopy

For fluorescence microscopy, excitation was performed using a mercury vapor lamp (HBO 100; Osram). A fluorescence filter-set with an excitation filter at 575/15 nm, BS593 beam splitter and emission filter at 624/40 nm was used. According to literature data [1], the Gd(EDTA) excitation is at 561 nm, and the emission is at 627 nm. A histogram of grey values was generated using ImageJ 1.53f Software (function: Analyze and PlotProfile).

Separation of particles

Particles from experimental set-ups with *T. pyriformis* in 1 % PPY medium containing 0.5 mM $GdCl_3$ after 24 h cultivation were used for separation. The particles at the bottom of the culture dish were first pulled off with a pipette and placed in a cell culture insert with a membrane of 8 µm pore size. By adding MQ water and constantly pipetting and mixing the solution, the particles can be separated from the cells through the pores of the cell culture insert. The flow-through was collected and centrifuged (4000 x g for 10 min). The supernatant was taken and discarded. The pellet was resuspended in 1 - 3 mL of MQ water. Storage of the separated particles was performed in MQ medium. Freshly separated particles were used for experiments, e.g. for reingestion experiments.

Supplementary Figures

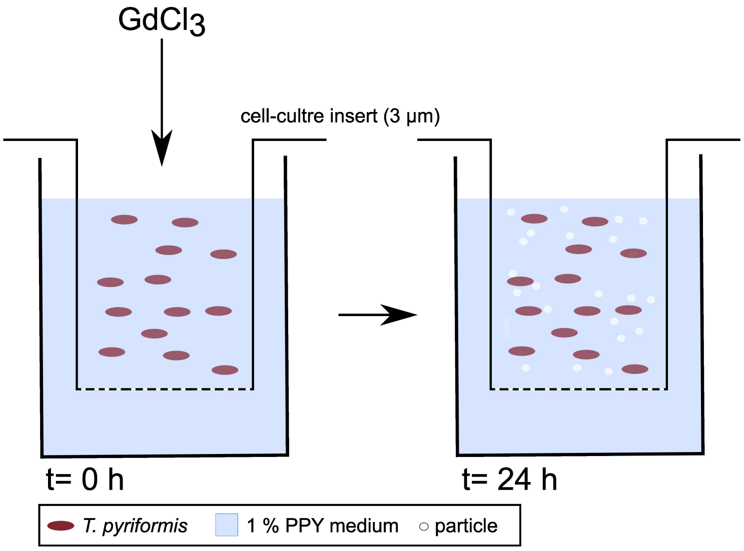

Figure S1: Experimental set-up to examine possible gadolinium-induced excretion of biomolecules that induce extracellular particle formation. The reaction volume (with 1 % PPY) is separated through a permeable cell-culture insert with a membrane with pores of about 3 µm to prevent diffusion of Gd-containing particles, but small biomolecules can pass. Cells of *T. pyriformis* are only in one compartment. Particles can be observed only in the compartment with the cells 24 h after adding Gd into both compartments. Thus, based on our results, an excretion of biomolecules inducing extracellular particle formation, as proposed, for example, in *T. thermophila* for Ag^+ ions [2], can be excluded in the case of Gd treatment.

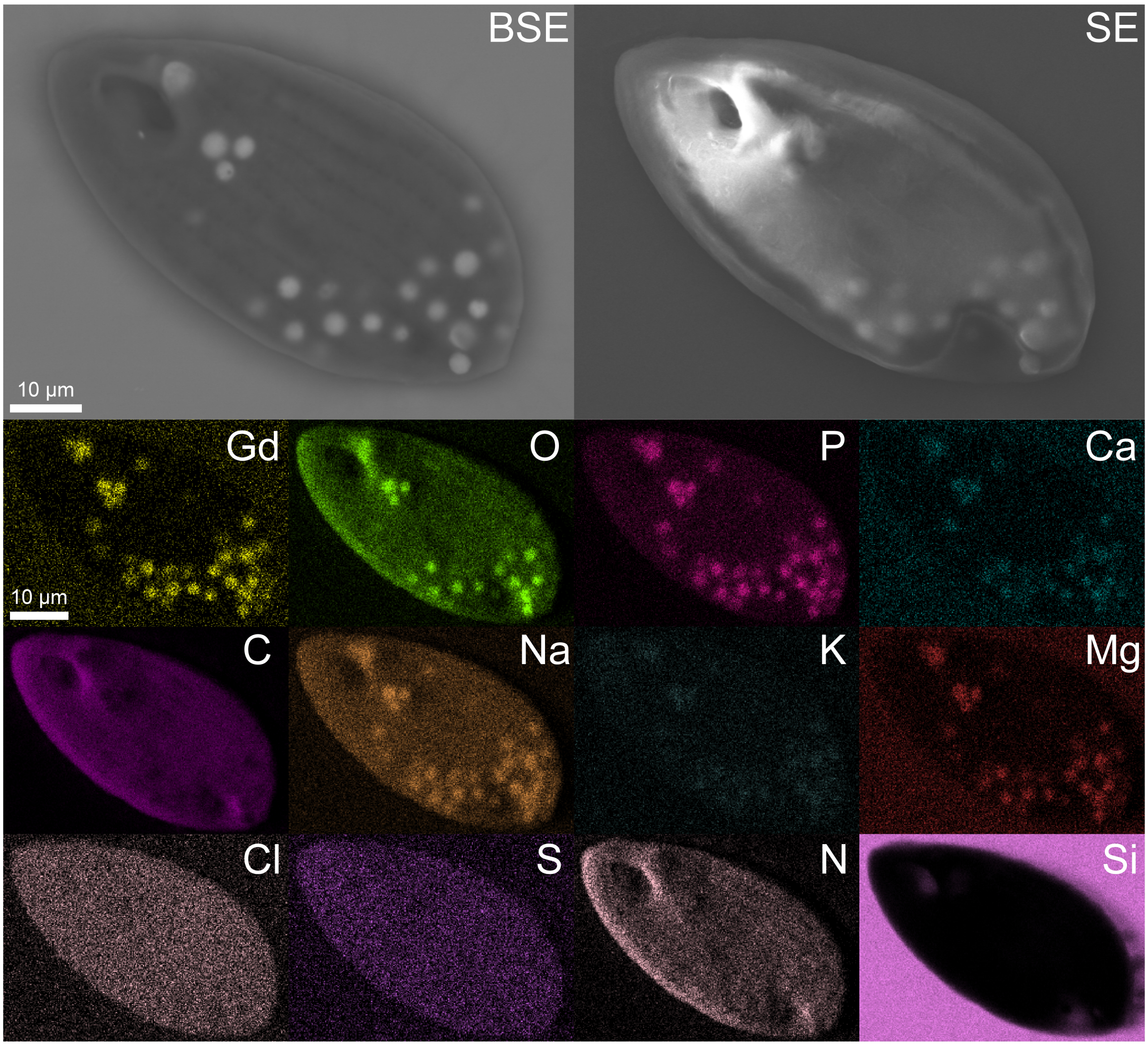

Figure S2: EDS-mapping of Gd treated cell of *T. pyriformis* in 1 % PP medium 24 h after treatment with 0.5 mM $GdCl_3$. Shown are the REM-BSE and -SE images of the cell, as well as detected signals for the elements Gd, O, P, Ca, C, Na, K, Mg, Cl, S, N, and Si. Especially the elements Gd, O, P, Ca, Na, K, and Mg are more concentrated in the particles formed than in the surrounding cell body. Si signal is due to the background.

Table S1: Overview of the influence of cultivation conditions on particle formation. Observation of particle formation, granule appearance and precipitate formation at different pH respectively $GdCl_3$ (and $CaCl_2$) concentrations along time after Gd treatment (- not observed to +++ high numbers observed; [1] granules containing Gd and Ca, [2] granules containing neither Gd nor Ca, [3] granules containing Ca and no Gd). Controls are approaches described in the respective row, always without Gd addition. *baseline calcium concentration in 1 % PP medium is about 0.16 mM.

parameters		particle formation inside cells	granule appearance inside cells		formation of precipitate in the medium
		Gd	Gd	control	Gd
0.5 mM $GdCl_3$ in MQ medium after 24 h with *T. pyriformis*		-	-	-	-
0.5 mM $GdCl_3$ in Y medium after 24 h with *T. pyriformis* (about pH 6)		-	-	-	-
0.5 mM $GdCl_3$ in 1 % PPY medium after 24 h with *T. pyriformis* (about pH 7)		+	-	-	-
0.5 mM $GdCl_3$ in 1 % PP after 24 h with *T. pyriformis*	pH 5	+	+ (1)	-	-
	pH 6	+	-	-	-
	pH 7	+	-	-	-
	pH 8	+	-	-	-
	pH 9	+	-	-	+
	pH 10	+	+ (3)	+ (2)	++
1 % PP medium at pH 7 after 24 h with *T. pyriformis*	0.025 mM $GdCl_3$	-	-		-
	0.05 mM $GdCl_3$	+	-		-
	0.25 mM $GdCl_3$	+	-		-
	0,5 mM $GdCl_3$	+	-		-
	1 mM $GdCl_3$	+	-		+
	2 mM $GdCl_3$	+	-		++
	3 mM $GdCl_3$	+	-		+++
	4 mM $GdCl_3$	cells die			+++
0.5 mM $GdCl_3$ at pH 7 after 24 h with *T. pyriformis*	1 % PP	+	-	-	-
	0.5 % PP	+	+ (1)	-	-
	0.25 % PP	+	+ (1)	-	-
1 mM $GdCl_3$ at pH 7 after 24 h (without cells)	1 % PP				+
	0.5 % PP				++
	0.25 % PP				+++
2 mM $GdCl_3$ at pH 7 after 24 h (without cells)	1 % PP				+
	0.5 % PP				++
	0.25 % PP				+++
0.5 mM $GdCl_3$ at pH 7 with *T. pyriformis*	7 days	+	+ (3)	+ (3)	-
1 % PP Medium (pH 7) 35 mM $CaCl_2$* and 0.5 mM $GdCl_3$ with *T. pyriformis* after 24 h		+	+ (3)	+ (3)	++
1 % PP Medium (pH 7) 35 mM $CaCl_2$* with *T. pyriformis* after 24 h		-		+ (3)	++

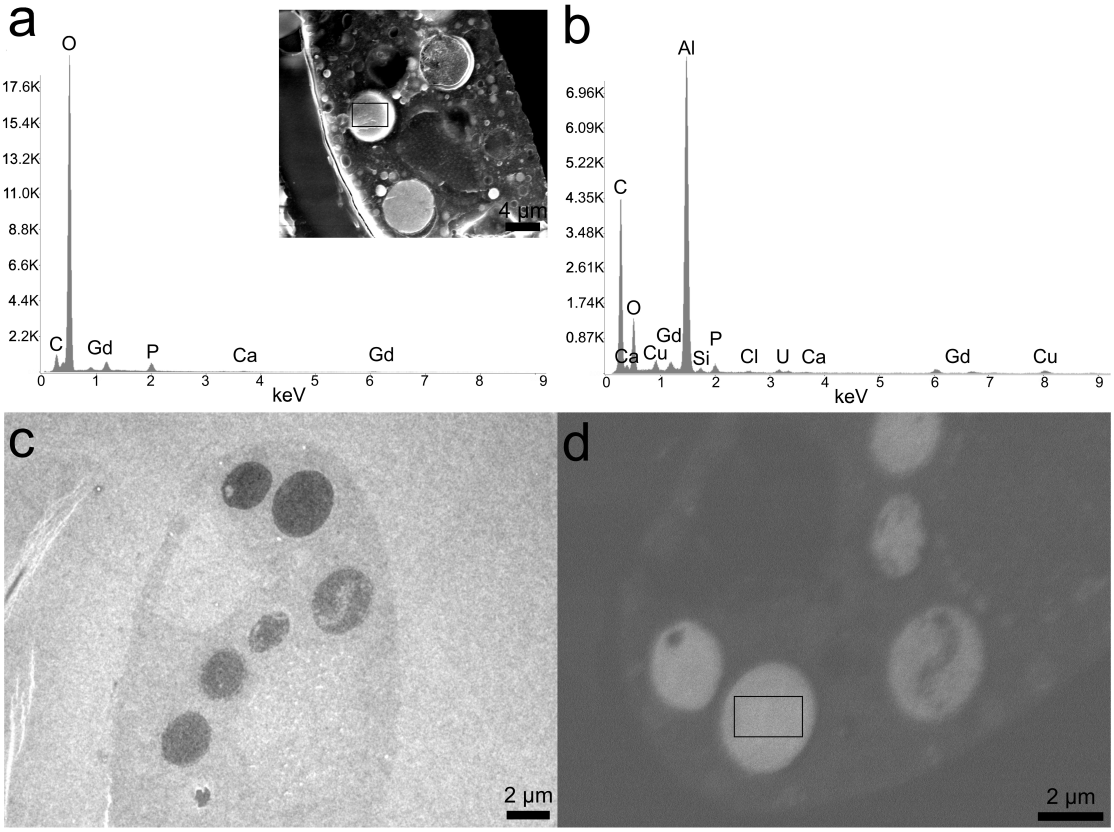

Figure S3: Elemental analysis of Gd-containing particles. a) EDS-spectra of Gd-containing particle in cryo freeze-fractured sample. The measured surface for EDS analysis is indicated in the insert. b) EDS-spectra of Gd-containing particle of a prepared ultra-thin section for TEM analysis (cell in c and d). The measured surface for EDS analysis is indicated in d. Elemental signals from Cu, Si, Al, and U can be traced back to the sample preparation, respectively, sample holder. c) TEM image of a cell of *T. pyriformis* 2 h after treatment with 0.5 mM $GdCl_3$ having dark-appearing Gd-containing particles. d) BSE-SEM of the same cell as in c. Note the different orientations of the cell.

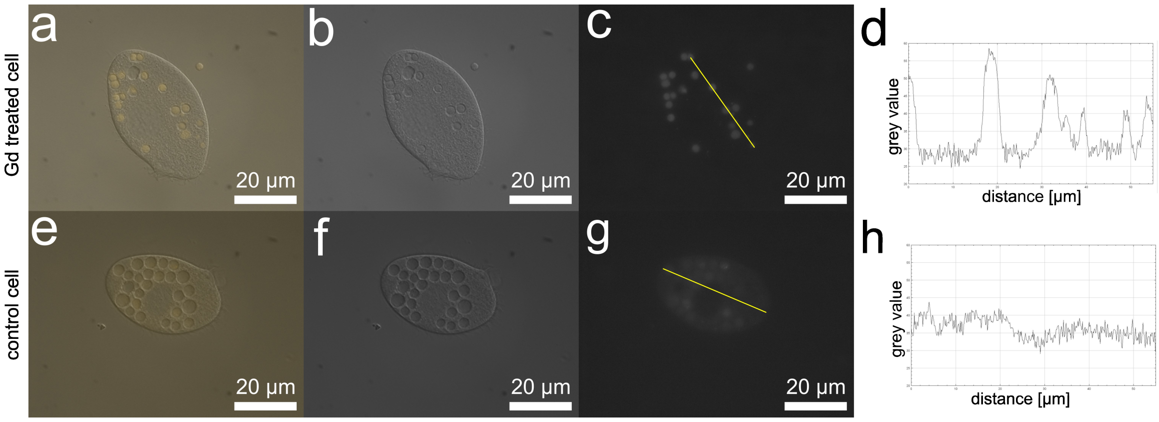

Figure S4: Fluorescence signals of Gadolinium in Gd-treated cells. a+e) Superimposed image of LM-DIC (light microscopic differential interference contrast) image and Gd-fluorescence-signal of Gd-treated (24 h after treatment with 0.5 mM GdCl3 in 1 % PPY medium) (a) and control (e) cells. b+f) LM-DIC image of Gd treated (b) and control (f) cell. c+g) Fluorescence image of Gd-signal of Gd treated (c) and control (g) cells. d+h) Histogram of the grey values of fluorescence signals over a measured distance in Gd treated (d) and control (h) cells. Particles in Gd treated cells show clearly more intense fluorescence signals than food vacuoles in control cells, confirming the gadolinous content of particles.

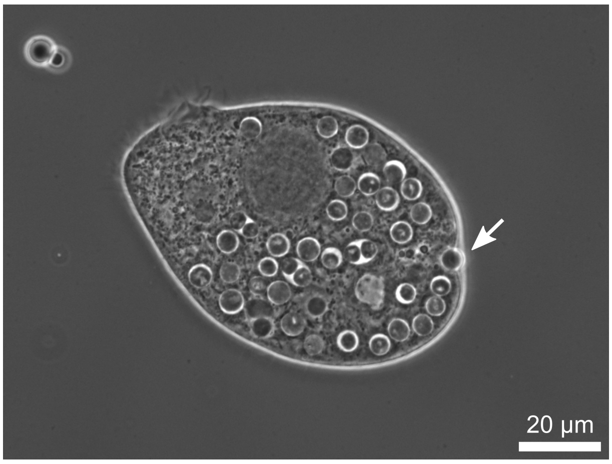

Movie S5: LM-Ph recording of a cell of *T. pyriformis* 5 h after treatment with 0.5 mM $GdCl_3$ in 1 % PPY medium. Time is given in seconds. The cell contains many Gd-containing particles. The pulsation of the contractile vacuole and the beating of the undulating membrane at the oral region are clearly visible. Excretion of a Gd-containing particle takes place from the second 17 at the cytopyge region at the posterior end of the cell.A frame of the video is shown here as an example.

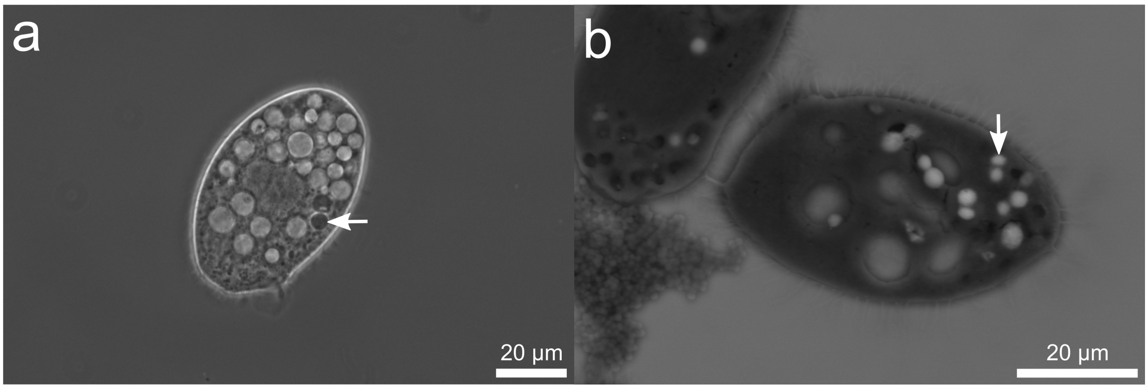

Figure S6: Reingestion of excreted and separated particles. Excreted Gd-containing particles from cells of *T. pyriformis* 24 h after treatment with 0.5 mM $GdCl_3$ in 1 % PPY medium were separated and offered to untreated cells. Observation after 24 h after particle treatment. a) LM-Ph image. Particles appear dark (arrow). b) BSE-REM image. Particles appear bright (arrow).

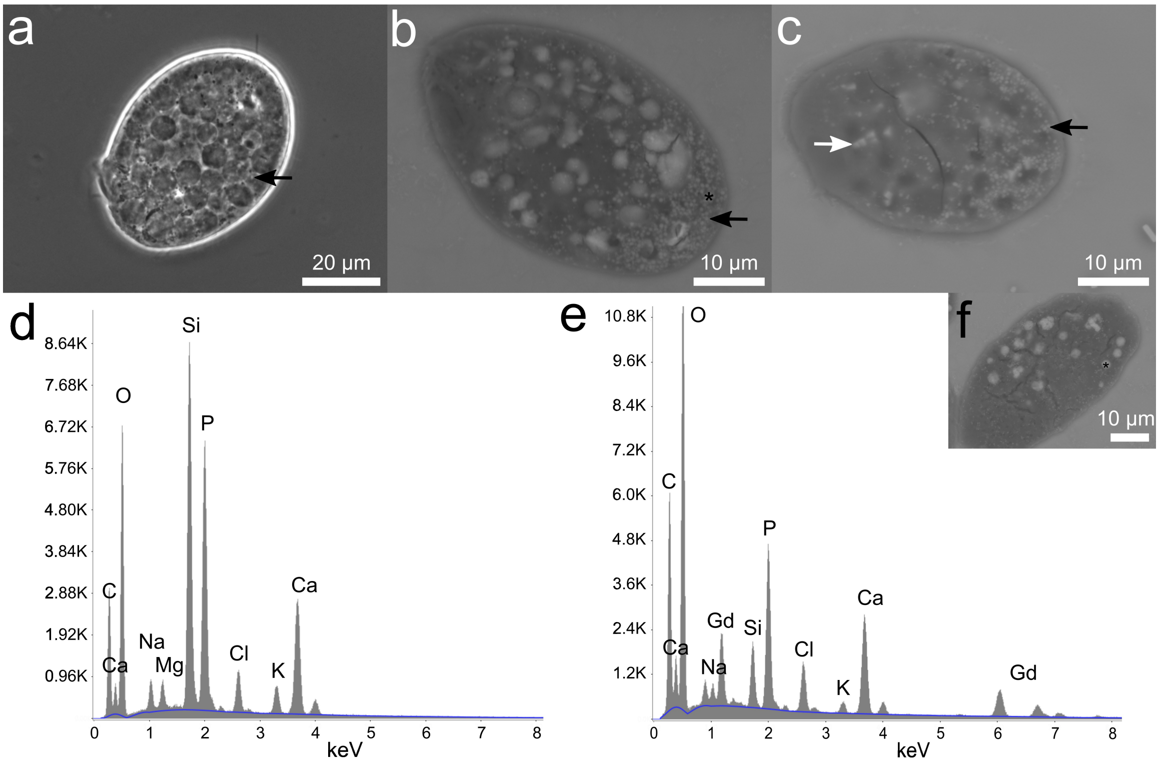

Figure S7: Formation of granules. a) LM-Ph image of *Tetrahymena pyriformis* 2.5 h after treatment with 0.5 mM $GdCl_3$ in 1 % PP medium at pH 5. Besides the dark-appearing particles inside the cell, many small light-refracting granules (arrow) are visible. b) SEM-BSE image of *Tetrahymena pyriformis* 24 h after treatment with 0.5 mM $GdCl_3$ and 35 mM $CaCl_2$ in 1 % PP medium at neutral pH. Besides Gd-containing particles also, small refracting granules (arrow), which are scattered all over the cell, can be observed. Star marks measurement point for EDS analysis in d. c) SEM-BSE image of *Tetrahymena pyriformis* 24 h after treatment with only 35 mM $CaCl_2$ in 1 % PP medium at neutral pH. The black arrow indicates small refracting granule, which are scattered all over the cell. Note that there is no particle formation as it was observed for cells with Gd treatment. White arrow indicates on NaCl crystal. d) EDS spectra of a granule of cell in b. e) EDS spectra of a Gd- and Ca-containing particle of the cell in f. In comparison with cells without additional treatment of $CaCl_2$ (Fig. 1, h), note the higher Ca signal in the particle. f) SEM-BSE image of *Tetrahymena pyriformis* 4 h after treatment with 0.5 mM $GdCl_3$ and 35 mM $CaCl_2$ in 1 % PP medium at neutral pH. Star marks measurement point for EDS analysis in e. The cell contains particles and granules.

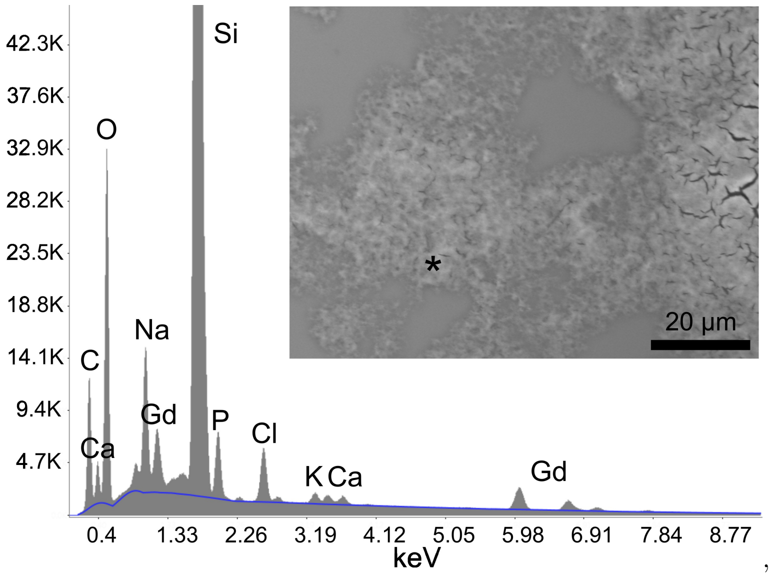

,

Figure S8: Formation of a precipitate. EDS spectra and SEM-BSE image of precipitate from the approach with 0.5 mM $GdCl_3$ in 1 % PP medium at pH 5. Star indicates the measurement point of EDS analysis. Note that the precipitate formed contains Gd.

Bibliography

[1] James J. Hagan, Susan Cicero. Taylor, and Michael F. Tweedle. “Fluorescence detection of gadolinium chelates separated by reversed-phase high-performance liquid chromatography”. en. In: *Analytical Chemistry* 60.6 (Mar. 1988), pp. 514–516. ISSN: 0003-2700, 1520-6882. DOI: 10.1021/ac00157a004. URL: https://pubs.acs.org/doi/abs/10.1021/ac00157a004 (visited on 10/22/2020).

[2] Katre Juganson et al. “Extracellular conversion of silver ions into silver nanoparticles by protozoan Tetrahymena thermophila”. en. In: *Environmental Science: Processes & Impacts* 15.1 (2013). Publisher: Royal Society of Chemistry, pp. 244–250. DOI: 10.1039/C2EM30731F. URL: https://pubs.rsc.org/en/content/articlelanding/2013/em/c2em30731f (visited on 03/17/2020).
